# COVID-19 and evolutionary medicine

**DOI:** 10.1093/emph/eoaa018

**Published:** 2020-06-16

**Authors:** Maciej Henneberg, Frank Rühli

**Affiliations:** e1 Biological Anthropology and Comparative Anatomy Unit, Adelaide Medical School, The University of Adelaide, Australia; e2 Institute of Evolutionary Medicine, University of Zurich, Switzerland

The coronavirus pandemic highlights the need for evolutionary perspectives. Evolutionary knowledge helps to address this disaster at many levels (Fig. 1), including at the level of the virus and in terms of human behaviour and our coevolutionary interactions with pathogens.

**Fig. 1:**
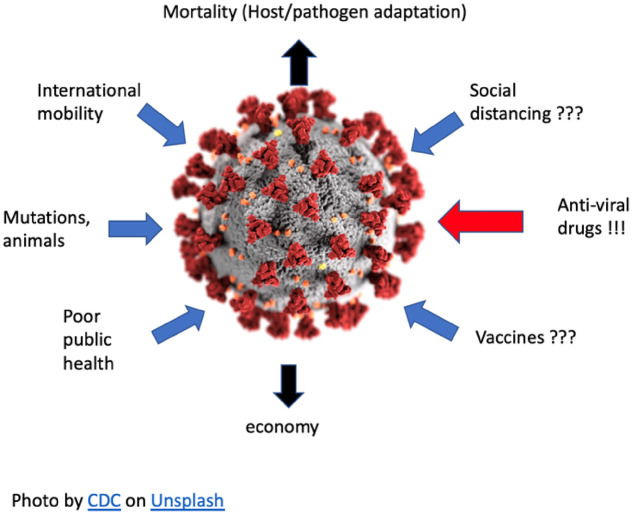
COVID-19 pandemics as a dynamic system.

## HUMAN BEHAVIOUR

Crises like the current pandemic allow us to see clearly our evolutionary legacy in terms of behaviour. Human innate conduct becomes obvious in crisis situations. It ranges from excessive altruism to extreme selfishness. Despite the rational underpinnings of governmental recommendations, people often do not follow minimal hygiene or social distancing. Human panic reactions are unconscious and irrational. They were formed by our earlier living conditions where flight-or-fight behaviours had adaptive value. Official rules often are not effective because of natural resistance towards orders that are counter-intuitive and constrain personal freedoms. We are not adapted to alter our routines quickly. This is why attempts at preventing the spread of infections will typically slow down transmission but are often unable to eliminate the pathogen.

Humans are not evolutionarily programmed for sustainable behaviour reaching beyond a short time horizon. Thus, planning reserves in the medical care and political system are counter-intuitive and, in conjunction with constant pressures on institutional economies, fails in crisis situations. People tend to forget and neglect catastrophes that occurred too far back in time. For example, in Switzerland, there is a concept of a *Katastrophenlücke* (‘disaster gap’, a term first used by C. Pfister, University of Berne, in relation to the lack of major natural disasters for more than 100 years). Old catastrophic situations tend to repeat themselves after generational memory fades.

## HOST–PATHOGEN COEVOLUTION AND HUMAN HISTORY

Microorganisms evolve quickly due to their short generation times and because point mutations are expressed easily. When new microorganisms evolve or are introduced to the human population, they may be highly virulent or transmissible, with further evolution shifting these parameters over time. A lot of infections are spread by human contacts. In the past, human interactions were usually limited to local communities and their immediate neighbours. Many pathogens that become pandemic are similar to gene flow, i.e., they require close physical contact for their spread. It has been estimated that to pass a gene (or an endemic pathogen) from China to South Africa would require more than 10 000 years in hunter–gatherer populations of our evolutionary past [1]. Thus, rapid pandemics are a modern phenomenon. This novel global transmission dynamic amplifies the consequences of infectious disease introductions.

A pathogen entering a local community elicits adaptive reactions. These are immune responses of individuals, and gene pool adaptations through fast-acting natural selection. Before the advent of effective preventive methods and therapies, opportunities for the operation of natural selection were very large—due to premature mortality, only about one-third of individuals born had an opportunity to pass their genes to the next generation [2]. Thus, adaptations of local gene pools to new pathogens were fast. Because pathogen transmission via human contact was slow, worldwide pandemics were rare.

The historically recorded pandemics were related to invasions at the end of Classical Antiquity and in the Middle Ages. They caused enormous numbers of deaths that destroyed economies and altered the biological characteristics of human populations (e.g. historical selective pressures for the *CCR5*-*Delta* 32 HIV-resistance allele). Public health measures in many of past pandemics were very similar to those introduced in the current pandemic, such as isolating sick individuals which was already described e.g. in the London Privy Council Rules and Orders [3]. Yet they were not effective because the only way to eradicate a pandemic—instead of allowing it to run its course and, through acquired immunity and natural selection to leave behind a decimated population not susceptible to the pathogen—is to find effective means of killing the pathogen or providing immunity to the population. To what extent the current pandemic can be compared to previous pandemics—such as the 1918 influenza pandemic—will continue to be debated as this pandemic’s course takes shape. Its dynamics is somewhat different for example due to the level of public and scientific awareness in the present situation.

Although the death toll of past pandemics was enormous, they did not kill everyone. One factor that has enabled humanity to persist involves a basic characteristic of populations: variation. Biological variability produced by mutation/selection balance, genetic polymorphisms, adaptive responses during ontogeny, life histories and particular ways of infections and immune responses results in different phenotypic characteristics that enable some individuals to survive pandemics. In the COVID-19 pandemic, the course of disease varies in different patients, from completely asymptomatic to fatal. Although initial clinical observations indicate characteristics of specific susceptibility such as age, sex and some co-morbidities, it is far from known to what extent these phenotypes reflect genetic variation, ontogenetic adaptability and particular characteristics of infection, such as timing of infection and viral load. Understanding the evolutionary drivers of these characteristics is of particular importance to public health, including because some of these traits may be enhancing existing health disparities.

Fast viral evolution makes it difficult for hosts to acquire lasting immunity to repeated infection, and not all organisms produce lasting immunity. Vaccines may therefore be only partly effective [4]. The only way to effectively eradicate COVID-19 is to remove its cause from bodies of individual patients and reduce contact with the (as yet unknown) natural reservoir. We need to develop an efficient medication that will stop reproduction of the virus in humans, and then use that medication in a way that will avoid evolution of resistance by the virus. Such ‘evolution proof’ solutions are another way that evolutionary medicine can contribute to stopping the current pandemic.

## A CALL FOR BROADER ACTION

Innovations appear randomly. One can foster a potentially innovative climate but not force an innovation. Thus, it is important to have free discussions and true collegiality in the research community, including across disciplines. At the moment many groups of medical scientists are working diligently and selflessly to control COVID-19, but their communications are limited and occur in the organizational structure of research institutions hampered by a multitude of complex regulations and informed by competition for commercial gain, grants and publication scores. Individual researchers in the process of doing research consider their incomes, professional careers and reputation. Governments who support medical research are concerned about appropriateness of their methods and quantities of support. Furthermore, humans did not evolve to cope with logical, mathematical problems. To understand exponential epidemiological data is beyond the primary abilities of human individuals, especially non-scientists making decisions in the current situation. All this slows the response of medical science to quickly arising global health threats.

At present (September 2020), no effective vaccines or cures are available. In an emergency, the complex machinery of medical science finds it difficult to become efficient and follow the principle of parsimony—cut out all the ‘fluff’ and go straight to the main aim. Only by taking evolutionary perspectives into account will we effectively solve the issue of the current and future pandemics. This must involve communication and collaboration across disciplines and an open mindset.

## FUNDING

Mäxi Foundation Switzerland (awarded to FR).

**Conflict of interest:** None declared.

## References

[1] Henneberg M. The gradual eurytopic evolution of humans: not from Africa alone In: Etty I (ed.). Man: Past, Present, and Future. Yogyakarta, Indonesia: Bigraf Publishing, 2001, 42–52.

[2] Rühli F Henneberg M. Biological future of humankind: ongoing evolution and the impact of recognition of human biological variation, Chapter 17 In: Tibayrenc M Ayala FJ (eds). On Human Nature. Amsterdam: Academic Press, 2016, 263–75.

[3] Hollyer B. (ed.). Privy Council Rules and Orders 1666 Reprinted in Bell WG 1924 The Great Plague in London. London: The Folio Society, 2001, 208–10.

[4] Innis BL Scorza FB Blum JS et al Meeting report: convening on the influenza human viral challenge model for universal influenza vaccines, Part 1: value; challenge virus selection; regulatory, industry and ethical considerations; increasing standardization, access and capacity. Vaccine 2019;37:4823–9. 31362819 10.1016/j.vaccine.2019.06.080 PMC6677912

